# Assessment of neutrophil gelatinase-associated lipocalin as a biomarker of acute kidney injury in acute heart failure patients

**DOI:** 10.1186/cc12363

**Published:** 2013-03-19

**Authors:** H Michalopoulou, H Michalopoulou, P Stamatis, J Xenogiannis, D Stamatis

**Affiliations:** 1 Metaxa Hospital, Athens, Greece

## Introduction

Growing evidence hints that bidirectional interaction between heart failure and kidney disease and renal insufficiency is a strong predictor of mortality as well as causally linked to the progression of heart failure. Neutrophil gelatinase-associated lipocalin (NGAL) is an early predictor of acute kidney injury (AKI). We evaluated the impact of NGAL on morbidity and mortality in patients with acute heart failure.

## Methods

Seventy-six patients presenting with symptoms consistent with acute heart failure (median age 72 years, 56% male) were enrolled. Plasma NGAL levels were measured by an ELISA at admission and compared with the glomerular filtration rate (eGFR) and B-natriuretic peptide (BNP) levels. The primary outcome was AKI development defined by RIFLE criteria (fall in GFR >25% or creatinine rise ≥50% from baseline, or a fall in urine output <0.53 ml/kg/hour) and secondary outcomes were duration of hospital stay and in-hospital mortality.

## Results

AKI developed in 11 patients (14.4%). The subgroup that during hospitalization developed renal dysfunction had increased NGAL values with respect to patients with preserved renal function (146 ± 42 vs. 84 ± 24 ng/ml, *P *0.05). NGAL significantly associated with admission eGFR (*r *= 0.72, *P *0.01) and BNP levels (*r *= 0.80, *P *0.01). For prediction of AKI, NGAL >1403 ng/ml has been related to renal insufficiency with sensitivity of 83% and specificity of 74% with area under the receiver operator curve of 0.70 (0.58 to 0.82). In the follow-up setting, NGAL was associated with recurrent hospital admission for heart failure (HR: 3.1, CI: 1.5 to 6.2) and cardiac death (HR: 2.2, CI: 1.8 to 4.5).

## Conclusion

NGAL is emerging as a promising biomarker of AKI in the setting of acute heart failure and elevated NGAL levels indicate a poor prognosis in this population regarding morbidity and mortality.

